# Oral Health-Related Quality of Life in Anticoagulated Patients with Warfarin Treatment: A Cross-Sectional Study

**DOI:** 10.3390/ijerph18073714

**Published:** 2021-04-02

**Authors:** Johana Alejandra Moreno-Drada, Alex Junio Silva da Cruz, Matheus Luis Soares de Faria, Luís Otávio Miranda Cota, Maria Auxiliadora Parreiras Martins, Isabela Almeida Pordeus, Mauro Henrique Nogueira Guimarães de Abreu

**Affiliations:** 1 School of Dentistry, Universidade Federal de Minas Gerais, Belo Horizonte CEP 31270-901, Brazil; junio.alex@hotmail.com (A.J.S.d.C.); matheuslsfaria@gmail.com (M.L.S.d.F.); luisotaviocota@gmail.com (L.O.M.C.); isabela.pordeus@gmail.com (I.A.P.); maurohenriqueabreu@gmail.com (M.H.N.G.d.A.); 2 Department of Pharmaceutical Products, Universidade Federal of Minas Gerais, Belo Horizonte CEP 31270-901, Brazil; auxiliadorapmartins@hotmail.com

**Keywords:** anticoagulants, oral health, quality of life, oral health-related quality of life

## Abstract

To evaluate factors associated with oral health-related quality of life (OHRQoL) in patients under oral anticoagulant therapy with warfarin, a cross-sectional study was conducted. Validated questionnaires assessed self-reported periodontal disease, demographic variables, and OHRQoL using the short version of the Oral Health Impact Profile (OHIP-14) instrument. After calibration (Kappa > 0.60), an examiner evaluated patients’ experience with dental caries and the need for dental prostheses. Statistical analysis involved proportions and measures of central tendency. Negative binomial regression models were used to estimate the rate ratios (RR) and the corresponding 95% confidence interval (CI). The sample consisted of 158 individuals, with a mean age of 58.8 years (SD = 12.1), of which 62.7% of the participants were women. The OHIP-14 mean was 10.62 (SD = 10.92). A higher OHIP-14 total score (worse OHRQoL) was associated with ethnic group, age, periodontal disease self-report, dental caries, and oral health self-report. Demographic and clinical factors can negatively influence the perception of anticoagulated patients on OHRQoL.

## 1. Introduction

Oral anticoagulant therapy (OAT) has increased in recent years [1,2], especially due to the increase in life expectancy [3]. OAT is recommended for outpatients with atrial fibrillation, prosthetic heart valves, venous thromboembolism, acute coronary syndrome or myocardial infarction, and pulmonary hypertension for prevention and treatment, reducing the risk of stroke-related morbidity and mortality [2,4,5,6,7,8]. Coumarin derivatives, such as warfarin, are the most commonly used drugs, especially in low and middle-income countries [6,9,10,11]. The efficacy and safety of warfarin depend on achieving the desired therapeutic range, monitored by the international normalized ratio (INR) [2,12]. Both insufficient and excessive anticoagulation effects can be harmful, increasing thromboembolic and hemorrhagic risks, respectively [2].

Although prior studies are scarce in the literature, oral health plays an essential role in individuals on oral anticoagulation. Meurman et al. [13] identified that part of the OAT patients had never brushed their teeth. The fear of gingival bleeding may well induce individuals to brush their teeth less frequently, increasing the number of plaque-covered teeth and causing deep gingival pockets [13,14]. Moreover, the population undergoing OAT tends to be older [3,15]. Other conditions increase their prevalence and severity in aged patients, such as periodontal disease, decreased salivary flow, and reduced host defenses, leading to different oral diseases. These conditions and insufficient knowledge of oral health may contribute to inadequate oral hygiene [14]. As a result, extractions in patients undergoing OAT are frequent [7,9,14]. The loss of teeth may also negatively influence essential oral functions [16].

The interest in Patient-Reported Outcome Measures (PROMs) in clinical research has been growing lately. The use of PROMs to assess the oral health related quality of life (OHRQoL) allows us to understand the impact of oral conditions on the subjects’ daily lives, a perspective that would probably not be achieved by clinical indicators [17]. Previous studies identified that the OHRQoL could be affected by sociodemographic factors, such as age, sex, ethnicity, and socioeconomic level [18,19,20]. Similarly, periodontal disease, teeth (decayed, missing, or filled), mucosal lesions, and denture problems were prevalent and showed a negative association with OHRQoL [19,21,22]. By contrast, a systematic review stated no consensus in the literature on the association between edentulism, caries, and periodontal conditions and OHRQoL [23]. Despite the increasing publications about this topic, there is limited knowledge about the behaviors, feelings, or repercussions of oral conditions in individuals undergoing OAT.

The risk of bleeding, dietary intake restrictions, and possible interactions of oral anticoagulants, such as warfarin, with other medications can interfere with daily activities in people undergoing OAT [8,24,25]. These events can affect the general quality of life of the population that uses oral anticoagulants. In addition to the uncertainties associated with OAT, there is an increased risk of oral bleeding due to oral diseases that can cause dissatisfaction and have a significant negative impact upon OHRQoL [14,25]. However, the scientific community has paid little attention to the interaction between complex long-term treatments, such as OAT and oral health care, and the impact oral health has on one’s quality of life [14,25]. Considering that most patients who receive oral anticoagulation suffer from chronic diseases that may be severe or life-threatening and undergo continuous drug treatment throughout their lives, the role of oral health and its impact on the quality of life in this population is unclear. Thus, this study aims to analyze the factors associated with OHRQoL in patients on OAT with warfarin.

## 2. Materials and Methods

The Ethics Committee for Human Research from Universidade Federal de Minas Gerais (UFMG) (logged under protocol number CAAE 17726219.0.0000.5149) approved the conduction of this study. Patients signed an informed consent form; participation was voluntary, and patients could refuse to answer any questions.

### 2.1. Participants and Data Collection

Outpatients from the Brazilian National Health System (SUS in Portuguese) undergoing oral anticoagulant treatment during 2019 at the Anticoagulation Clinic of the Hospital das Clínicas of UFMG were evaluated. The anticoagulation clinic is multidisciplinary, with established protocols for patient education and dose adjustments [11]. A consecutive sample of patients was selected. The patients under medical treatment at the Anticoagulation Clinic of the Hospital das Clínicas of UFMG and those who were eligible and gave authorization were recruited consecutively. The sample calculation was performed with a 95% confidence level (CI) and an 80% power and the comparison of means on the information about the impact of untreated dental caries on the quality of life, considering the study of De Oliveira et al. [26], resulting in a minimum of 120 patients for the sample size. Considering the possibility of missing data for some variables, we added 30% to this sample size, resulting in a minimum of 156 patients.

This study included patients of ≥18 years of age on oral anticoagulation with warfarin. Exclusion criteria were: patients with insufficient information in their medical history, individuals that could not communicate their answers to the questions asked, or patients with spontaneous bleeding before the dental examination.

Data were collected from October 2019 to February 2020. Each patient was asked to answer a structured questionnaire with a combination of sociodemographic questions: the Brazilian version of the Oral Health Impact Profile (OHIP-14) [26], which has been designed to measure the perception of the social impact of oral disorders [27,28], together with the periodontal disease self-report instrument [29].

### 2.2. Instruments

The self-reported periodontal disease instrument evaluated sociodemographic information, risk factors for periodontal disease, self-reported oral health measures, and periodontal status. The preferred clinical examination is periodontal probing, and although it remains the gold standard for the diagnosis of periodontitis [29], it was not considered in this group of patients. The explanation is given because many patients on warfarin have heart diseases [30] or prosthetic heart valves with a risk for infective endocarditis [31], treated with the recommendation of antibiotic prophylaxis before dental procedures involving the manipulation of the gingival tissue, the periapical region of the teeth, or perforation of the oral mucosa [32]. The clinical evaluation would imply a risk of gingival bleeding that is difficult to control or a risk of endocarditis, in addition to the need to implement prophylactic antibiotic therapy. Moreover, the self-reported periodontal disease instrument has shown good accuracy in identifying sick individuals, especially those with severe periodontitis. The sensitivity and specificity showed a moderate to good value in identifying periodontal disease in different predictive models [29].

The OHIP-14 is a specific indicator of OHRQoL, which demonstrated good reliability and validity [28]. The OHIP-14 items involved the evaluation of seven conceptual subscales: functional limitation, physical pain, psychological distress, physical disability, psychological disability, social disability, and handicap [26]. The dependent variable analyzed in this study was the OHIP-14, with 14 questions and an answer possibility from 0 to 4. The total score range was 0 to 56, where the highest OHIP-14 score indicated a worse quality of life. The mean and median values of OHIP-14 were calculated for each covariate.

### 2.3. Face-to-Face Interviews and Clinical Examination

Trained researchers conducted face-to-face interviews. Prior to the fieldwork, an examiner underwent a training and calibration exercise to diagnose dental caries. Calibration was carried out using images from different clinical situations on two separate occasions. A pilot study was conducted before the fieldwork, where ten adult patients were evaluated at the School of Dentistry at Universidade Federal de Minas Gerais. The calibration level was observed through a Cohen’s Kappa coefficient above 0.60, which is considered adequate. A clinical examination was performed using DMFT index (decayed, missing, and filled teeth index) to assess the patients’ caries experience. Its value was expressed by the sum of decayed, missing, and filled teeth to obtain an individual index. The use or not of prostheses and the type of prostheses were clinically established [33].

### 2.4. Statistical Analysis

This analysis involved descriptive analysis, together with the calculation of proportions and central tendency and variability measures. Negative binomial regression models were used to estimate the unadjusted and adjusted rate ratios (RR) and corresponding 95% CI. First, unadjusted negative binomial regression models were carried out to estimate unadjusted RR (95% CI) and *p*-values for each of the 35 covariates included one by one. In this first step, any covariate with a *p*-value of less than 0.25 was a candidate to be tested in the final adjusted negative binomial regression model. As the focus of interest was on the independent effects of each covariate, all potential variables were included in the unadjusted model, which had age, sex, self-reported skin color, place of residence, smoking, tooth brushing frequency, gum disease, tooth migration, oral health, periodontal surgery, decayed (DMFT index) tooth indicated for extraction, and need for a prosthesis. Only covariates with a *p*-value lower than 0.05 were maintained in the final model when all variables would be presented together. The ratio between residual deviance and degree of freedom, as well as results from the chi-squared test of the residual deviance, was indicated to evaluate the goodness of fit of the final model [34,35]. Missing data were not computed in the statistical analysis. Variation Inflation Factor (VIF) was calculated for multicollinearity diagnosis. Statistical analyses were carried out in SPSS statistics software Version 24 (IBM, Chicago, IL, USA).

## 3. Results

The surveyed sample included 158 patients. The mean age (SD) was 58.8 (12.0) years, with a predominance of the female sex (62.7%), and 26.6% of the individuals reported that their skin color was white. We found ten patients who reported being smokers. The range number of cigarettes per day was 7 to 20, with a mean of 7.2 and a standard deviation of 5.96.

Regarding the indications for OAT, the results showed that the majority of patients had some fibrillation (19.7% non-valve atrial fibrillation/flutter and 23.7% valve atrial fibrillation/flutter) or some prosthetic heart valves (4.6% with biological valve prosthesis (mitral), 27.0% with mechanical valve prosthesis (aortic) and 34.2% with mechanical valve prosthesis (mitral)). The sum of these diagnoses was higher than 100%, considering that the same patient could have more than one OAT indication.

The mean of the years of school of the included patients was 6.63 (±3.87). The mean OHIP-14 of the sample was 10.6 (±10.9). The descriptive characteristics of the patients included in this study are presented in Table 1.

The final multivariate-adjusted model consisted of ten variables. Individuals from other races were more likely to have poor OHRQoL than white individuals (RR = 1.047; 95% CI 1.024–1.070). Older individuals were less likely to have poor OHRQoL (RR = 0.998; 95% CI 0.997–0.999). Patients who reported poor or fair oral health were more likely to have poor OHRQoL (RR = 1.055; 95% CI 1.028–1.083) than those who reported excellent, very good, or good oral health. Patients who reported gingival disease (RR = 1.031; 95% CI 1.013–1.048), dental migration (RR = 1.034; 95% CI 1.011–1.057), scaling and root planing (RR = 1.027; 95% CI 1.006–1.048), and periodontal surgery (RR = 1.032; 95% CI 1.010–1.055) had poor OHRQoL. Likewise, the individuals that presented more decayed (RR = 1.006; 95% CI 1.003–1.008), missing (RR = 1.002; 95% CI 1.001–1.004), and filled teeth (RR = 1.004; 95% CI 1.002–1.006) also had poor OHRQoL (Table 2). Parameters of the goodness of fit were adequate. The chi-squared test of the residual deviance results, with a *p*-value equal to 0.997, and the ratio between residual deviance and degree of freedom resulted in 1.078, indicating that the model fit well. VIF values were all lower than 10, showing no problem with collinearity.

## 4. Discussion

We found that the OHRQoL is associated with some sociodemographic factors, periodontal self-reported factors, and other clinical factors. White and older individuals were less likely to have poor OHRQoL. Patients who reported gingival disease, dental migration, scaling and root planing, periodontal surgery, poor or fair oral health, and more decayed, missing, and filled teeth had poor OHRQoL.

Almost half of the OHIP-14 value refers to the dimensions of psychological discomfort and pain. We must bear in mind that most oral problems do not represent an immediate risk of death; however, they are responsible for reducing the quality of life of individuals, prolonging pain states, causing functional, aesthetic, and nutritional problems, and due to everything above, leading to psychological issues [36]. High levels of stress, anxiety, and depression have been reported in patients with dental pain in the literature, reducing the quality of life [37]. 

Some authors have discussed the relationship between ethnic groups and OHRQoL. White individuals tend to show better oral status than other ethnic groups. This situation occurs because they tend to exhibit the highest social grade [38,39,40]. Ethnicity was the only social factor associated with OHRQoL in our study, which is different from that observed in other studies [18,19]. This is possibly because the present study was performed in a public hospital where most of the population presented low income and education levels.

Oral health problems have become a significant issue in old age. These problems include tooth loss, edentulism, clinical attachment loss, coronal and root caries, oral mucosa lesions, the use of non-functional dental prostheses (partial or total), chewing problems, among other conditions [21,41]. As seen in other studies, we expected a greater impact on OHRQoL in older people [18,42]; however, our findings showed the opposite. Some feasible explanations prove that dental esthetics is related to a person’s self-esteem and social interaction. Older adults seem more likely to be satisfied with their dental appearance than younger individuals [43]. Since dental esthetics may not be of primary concern among elders, this fact does not necessarily impact or impact the OHRQoL less in this age group. Moreover, dental perceptions are subjective and are associated with cultural characteristics and personality profiles [44]. This result was expected due to a variance in OHIP-14 scores, depending on the societal values where the construct was applied.

Severe periodontitis has been related to the OHRQoL [45,46,47,48]. The literature showed that the difference in OHIP-14 scores for patients with a probing depth > 6 mm was statistically more significant than for patients with a probing depth < 6 mm. Likewise, periodontal surgery was a statistically significant predictor for the OHIP-14 score [45]. Similarly, this study demonstrated the importance of gingival disease, tooth migration, scaling and root planing, and periodontal surgery related to OHRQoL. Moreover, the self-reported periodontal disease instrument allows one to understand the perception that individuals have regarding their oral and periodontal health. This perspective could be relevant to understand the impact of oral conditions on one’s quality of life. It is important to reinforce that a high percentage of patients in our study would need antibiotic prophylaxis before clinical examination considering their health condition [30,31,32]. In this clinical situation, the utility of self-report measures for periodontal disease is once more justified.

Untreated dental caries, poor self-perception of oral health, and tooth loss have already been associated with poor OHRQoL [27,49,50]. Even a meta-analysis reported that tooth loss is associated with unfavorable OHRQoL scores, regardless of the study location and the OHRQoL instrument [49]. These are consistent with our findings. Individuals with untreated dental caries may experience pain, mouth infection, and tooth loss, affecting adults’ productivity at work, social interaction, and other daily activities. Furthermore, untreated caries represent a significant social, biological, and financial burden on individuals [51], negatively impacting OHRQoL and needing to be controlled using a population approach [52].

Our study identified that patients using warfarin have factors associated with OHRQoL, similar to the general population, as shown by a systemic review. This review indicates that different studies reported poor OHRQoL in individuals with tooth loss, periodontal disease, and dental caries in the general population [53]. Even though our research did not analyze a group without OAT, we consider that this study provides information on the impact of oral health on the daily life of patients undergoing OAT. Despite the fact that taking warfarin was not a covariate in our study, future investigations that allow such a comparison are recommended. Some tested covariates were not associated with OHRQoL. Alcohol consumption, smoking, and diabetes were not associated with OHRQoL. Associations of these variables and quality of life have been reported in the literature; however, these associations are directed to alcohol abuse [54], impaired glycemic control or complications of diabetes [55], and the number of cigarettes smoked [56]. In our study, the details of these variables were not analyzed according to the amount of alcohol or cigarettes, nor were glycemic control or diabetes complications analyzed.

Subjective perceptions linked to culture and personality [44] may have altered the association of OHRQoL with sex and dental prostheses. Some studies related that women had a poorer OHRQoL compared to men [18,42], most likely explained by the fact that women are more affected by their physical appearance [57]. On the other hand, OHRQoL has been better in people who use dental prostheses than those who do not use and/or need them [19,57,58,59]. However, this study found no associations between sex or dental prostheses and OHRQoL. Perhaps, similar to age, dental esthetics played an essential role in the non-association of these variables. For this sample on oral anticoagulation, dental esthetics may not have been a primary concern. Nevertheless, most of the partial dentate and edentulous rehabilitated subjects used removable prostheses in the analyzed sample. A prior study reported that removable prostheses did not constitute an essential factor in determining good or bad OHRQoL [60]. Furthermore, a systematic review noted that in longer follow-ups (>9 months), removable partial dentures did not improve OHRQoL [61].

Some limitations of this study need to be acknowledged. First, this study uses a cross-sectional design, and the temporal relationship and causality between the outcome and the exposure cannot be determined. In this way, it cannot be guaranteed that the exposure preceded the outcome because there was no follow-up. In other words, it will be difficult to determine whether a particular factor is a cause or the result of another element. Longitudinal studies are needed to assess the temporality and causality of exposure [62,63]. Our sample is not selected at random, and our results have no external validity. Moreover, despite the fact that some values of RR are statistically significant, their effect on OHRQoL could be considered small. Despite the limitations, some strengths should also be highlighted. This study provides information on the impact of oral health on the daily life of patients undergoing OAT. This information can contribute to the decisions of public policymakers regarding the oral health of the population using oral anticoagulants. The impact of OHRQoL was also evaluated in a group of patients undergoing chronic medical treatment, and this evaluation is not common in the literature. Therefore, we recommend conducting future longitudinal research that improves the knowledge of oral conditions and the OHRQoL of this population. This study expands one’s understanding of the impact that oral diseases and other variables have on the OHRQoL of anticoagulated patients. It is important to highlight the importance of an interdisciplinary team focused on a comprehensive approach to the patient with systemic pathologies. This information, which is a rare topic of discussion in the literature, may well be useful when organizing oral health care in this growing population.

## 5. Conclusions

Demographic and oral health condition factors can influence poor OHRQoL among anticoagulated patients with warfarin. Oral health and psychological factors are important for a better OHRQoL in this type of increasing population. 

## Figures and Tables

**Table 1:** Characteristics of the patients under anticoagulation therapy with warfarin, Belo Horizonte, Brazil, 2019–2020.

Quantitative Variables	Mean (SD)	Minimum–Maximum
OHIP14- Oral Health Impact Profile (*N* = 158)	10.62 (10.92)	0–53
Functional limitation (*N* = 158)	1.35 (2.03)	0–8
Physical pain (*N* = 158)	2.48 (2.35)	0–8
Psychological discomfort (*N* = 158)	2.35 (2.70)	0–8
Physical disability (*N* = 158)	1.53 (2.19)	0–8
Psychological disability (*N* = 158)	1.52 (2.07)	0–8
Social disability (*N* = 158)	0.54 (1.30)	0–8
Handicap (*N* = 158)	0.85 (1.59)	0–8
Age (*N* = 158)	58.80 (12.05)	27–91
Years of school (*N* = 158)	6.63 (3.87)	0–20
INR-International normalized ratio (*N* = 157)	2.61 (0.82)	1.06–5.40
Number of teeth self-report (*N* = 154)	14.94 (11.02)	0–36
DMFT index- Decayed, missing, and filled teeth index (*N* = 158)	22.92 (7.57)	1–32
Decayed (DMFT index) (*N* = 158)	1.10 (1.85)	0–18
Missing (DMFT index) (*N* = 158)	16.23 (10.96)	0–32
Tooth indicated for extraction (*N* = 158)	0.27 (1.45)	0–17
Filled teeth (DMFT index) (*N* = 158)	5.43 (5.75)	0–24
**Categorical Variables**	**Frequency**	**%**
Sex (*N* = 158)		
Male	59	37.3
Female	99	62.7
Skin color (*N* = 158)		
White	42	26.6
Others	116	73.4
Place of residence (*N* = 158)		
Belo Horizonte	83	52.5
Small cities near Belo Horizonte	75	47.5
Education level (*N* = 158)		
Up to 8 years of formal education	96	60.8
More than 8 years of formal education	62	39.2
Household Income (*N* = 158)		
<1 Minimum wage	11	7.0
>1 Minimum wage	147	93.0
Alcohol consumption (*N* = 158)	22	13.9
Smoking (*N* = 158)	13	8.2
Diabetes (*N* = 157)	21	13.4
Dental flossing (*N* = 158)	93	58.9
Dental flossing frequency (*N* = 93)		
Occasionally	44	47.3
Daily	49	52.7
Tooth brushing frequency (*N* = 158)		
Once a day	10	6.3
Twice a day or more	148	93.7
Last dental checkup (*N* = 157)		
Up to 6 months	61	38.9
1 year or more	96	61.1
Gum disease (*N* = 125)	37	29.6
Tooth migration (*N* = 123)	46	37.4
Tooth mobility (*N* = 125)	38	30.4
Tooth loss (*N* = 124)	13	10.5
Oral health (*N* = 157)		
Excellent/Very Good/Good	114	72.6
Poor/Fair	43	27.4
Scaling and root planing (*N* = 124)	87	70.2
Periodontal surgery (*N* = 125)	10	8.0
Bone loss (*N* = 125)	24	19.2
Use of maxillary prostheses (*N* = 158)	84	53.2
Use of mandibular prostheses (*N* = 158)	51	32.3
Need for general prostheses (*N* = 158)		
Needs maxillary and mandibular prostheses	71	44.9
Needs only maxillary prosthesis or only mandibular prosthesis	43	27.2
None required	44	27.8
Need for maxillary prostheses (*N* = 158)	80	50.6
Need for mandibular prostheses (*N* = 158)	105	66.5

Minimum wage in Brazil is BRL 1100.00 (Real), which is approximately equivalent to USD 200.00 (American Dollar).

**Table 2:** Factors associated with oral health related quality of life among patients under anticoagulation therapy with warfarin. Belo Horizonte, Brazil, 2019–2020.

Variable	OHIP-14 Scores (Mean; Median)	Unadjusted Rate Ratio (95% CI)	*p* Value	Adjusted Rate Ratio (95% CI)	*p* Value
Sex (*N* = 158)					
Male (*N* = 59)	6.2; 4.0	1	<0.001		
Female (*N* = 99)	10.3; 9.0	1.079 (1.036–1.126)			
Skin color (*N* = 158)					
White (*N* = 42)	5.9; 4.0	1	0.004	1	<0.001
Others (*N* = 116)	12.3; 8.0	1.081 (1.025–1.142)		1.047 (1.024–1.070)	
Age (*N* = 158)		0.998 (0.997–0.999)	<0.001	0.998 (0.997–0.999)	<0.001
INR- International normalized ratio (*N* = 157)		0.999 (0.978–1.019)	0.895		
Place of residence (*N* = 158)					
Belo Horizonte (*N* = 83)	9.4; 7.0	1	0.140		
Small cities near of Belo Horizonte (*N* = 75)	11.9; 8.0	1.021 (0.993–1.049)			
Education level (*N* = 158)					
Up to 8 years of formal education (*N* = 96)	10.5; 7.0	1	0.808		
More than 8 years of formal education (*N* = 62)	10.9; 9.0	1.003 (0.976–1.031)			
Household Income (*N* = 158)					
<1 Minimum wage (*N* = 11)	10.9; 6.0	1	0.945		
>1 Minimum wage (*N* = 147)	10.6; 7.0	0.998 (0.930–1.069)			
Alcohol consumption (*N* = 158)					
No (*N* = 136)	10.5; 7.0	1	0.826		
Yes (*N* = 22)	11.2; 8.0	1.005 (0.961–1.051)			
Smoking (*N* = 158)					
No (*N* = 145)	11.2; 8.0	1	0.005		
Yes (*N* = 13)	4.1; 4.0	0.878 (0.801–0.962)			
Diabetes (*N* = 157)					
No (*N* = 136)	10.6; 7.0	1	0.756		
Yes (*N* = 21)	11.3; 9.0	1.005 (0.973–1.039)			
Dental flossing (*N* = 158)					
No (*N* = 65)	8.0; 6.0	1	0.010		
Yes (*N* = 93)	12.4; 9.0	1.040 (1.009–1.073)			
Dental flossing frequency (*N* = 158)					
Occasionally or not use (*N* = 109)	10.1; 6.0	1	0.345		
Daily (*N* = 49)	11.8; 9.0	1.013 (0.986–1.040)			
Tooth brushing frequency (*N* = 158)					
Once a day (*N* = 10)	10.3; 7.0	1	0.926		
Twice a day or more (*N* = 148)	10.6; 7.0	1.003 (0.944–1.065)			
Last dental checkup (*N* = 157)					
Up to 6 months (*N* = 61)	10.3; 7.0	1	0.739		
1 year or more (*N* = 96)	10.9; 7.0	0.995 (0.968–1.023)			
Gingival disease (*N* = 125)					
No (*N* = 88)	8.6; 6.0	1	<0.001	1	0.001
Yes (*N* = 37)	17.0; 13.0	1.054 (1.026–1.083)		1.031 (1.013–1.048)	
Dental migration (*N* = 123)					
No (*N* = 77)	7.8; 6.0	1	<0.001	1	0.003
Yes (*N* = 46)	16.9; 16.5	1.066 (1.034–1.098)		1.034 (1.011–1.057)	
Tooth mobility (*N* = 125)					
No (*N* = 87)	9.3; 6.0	1	0.003		
Yes (*N* = 38)	15.7; 12.5	1.013 (1.013–1.069)			
Tooth loss (*N* = 124)					
No (*N* = 111)	10.9; 7.0	1	0.364		
Yes (*N* = 13)	12.8; 9.0	1.013 (0.985–1.042)			
Oral health (*N* = 157)					
Excellent/Very Good/Good (*N* = 114)	7.0; 5.0	1	<0.001	1	<0.001
Poor/Fair (*N* = 43)	20.4; 19.0	1.090 (1.061–1.120)		1.055 (1.028–1.083)	
Scaling and root planing (*N* = 124)					
No (*N* = 37)	9.1; 6.0	1	0.174	1	0.011
Yes (*N* = 87)	12.2; 9.0	1.026 (0.989–1.064)		1.027 (1.006–1.048)	
Periodontal surgery (*N* = 125)					
No (*N* = 115)	10.6; 7.0	1	0.003	1	0.004
Yes (*N* = 10)	18.0; 21.0	1.036 (1.012–1.061)		1.032 (1.010–1.055)	
Bone loss (*N* = 125)					
Yes (*N* = 24)	14.2; 10.0	1	0.074		
No (*N* = 101)	10.5; 7.0	1.023 (0.998–1.049)			
Number of teeth self-reported (*N* = 154)		1.001 (1.000–1.002)	0.188		
Use of maxillary prostheses (*N* = 158)					
No (*N* = 74)	10.8; 8.0	1	0.859		
Yes (*N* = 84)	10.5; 6.0	0.998 (0.971–1.026)			
Use of mandibular prostheses (*N* = 158)					
No (*N* = 107)	11.6; 8.0	1	0.107		
Yes (*N* = 51)	8.5; 6.0	0.972 (0.939–1.006)			
Need for general prostheses (*N* = 158)					
Needs maxillary and mandibular prostheses (*N* = 71)	12.7; 8.0	1			
Needs only maxillary prosthesis or only mandibular prosthesis (*N* = 43)	10.0; 8.0	0.981 (0.951–1.013)	0.238		
None required (*N* = 44)	7.9; 4.5	0.957 (0.919–0.997)	0.035		
Need for maxillary prostheses (*N* = 158)					
No (*N* = 78)	9.1; 6.0	1	0.091		
Yes (*N* = 80)	12.1; 8.0	1.024 (0.996–1.054)			
Need for mandibular prostheses (*N* = 158)					
No (*N* = 53)	7.8; 6.0	1	0.029		
Yes (*N* = 105)	12.1; 8.0	1.042 (1.004–1.081)			
DMFT index-Decayed, missing, and filled teeth index (*N* = 158)		1.000 (0.998–1.001)	0.788		
Decayed (DMFT index) (*N* = 158)		1.004 (1.003–1.005)	<0.001	1.006 (1.003–1.008)	<0.001
Missing (DMFT index) (*N* = 158)		0.999 (0.998–1.000)	0.190	1.002 (1.001–1.004)	0.007
Tooth indicated for extraction (*N* = 158)		1.004 (1.003–1.005)	<0.001		
Filled Teeth (DMFT) (*N* = 158)		1.001 (0.999–1.003)	0.190	1.004 (1.002–1.006)	<0.001

Minimum wage in Brazil is BRL 1100.00 (Real), which is approximately equivalent to USD 200.00 (American Dollar).

## Data Availability

All data and materials, as well as a software application or custom code, support the published claims and comply with field standards.
